# Association between vaccination and myasthenia gravis: a systematic review and meta-analysis

**DOI:** 10.3389/fimmu.2026.1739730

**Published:** 2026-02-11

**Authors:** Chang Guan, Ruonan Zhang, Peixi Zhao, Ying Zhang, Lan Yu, Huijing Cui, Li Jiang, Tong Wu, Fang Liu, Yang Wu, Lin Huang, Hongmei Nan, Jian Wang, Peng Xu

**Affiliations:** 1 College of Traditional Chinese Medicine, Changchun University of Traditional Chinese Medicine, Changchun, Jilin, China; 2 Department of Neurology, The Affiliated Hospital of Changchun University of Chinese Medicine, Changchun, Jilin, China; 3 Department of Neurology, Nanguan District Traditional Chinese Medicine Hospital, Changchun, Jilin, China; 4 Traditional Chinese Medicine Department, Nanguan District Traditional Chinese Medicine Hospital, Changchun, Jilin, China; 5 College of Traditional Chinese Medicine, Zhejiang Chinese Medical University, Hangzhou, Zhejiang, China

**Keywords:** autoimmunity, meta-analysis, myasthenia gravis, systematic review, vaccination

## Abstract

**Background:**

Myasthenia gravis (MG) is a rare autoimmune disorder characterized by fluctuating muscle weakness due to impaired neuromuscular transmission. Vaccination remains a cornerstone of infectious disease prevention, yet concerns persist regarding potential autoimmune exacerbation in susceptible individuals. This systematic review and meta-analysis aimed to synthesize available evidence on the association between vaccination and MG, evaluating both vaccine effectiveness and safety in this population.

**Methods:**

Observational studies in cohort or case-control formats were identified through systematic searches of PubMed, Web of Science, Embase, Cochrane Library, SinoMed, CNKI, Wanfang, and VIP databases from inception to June 24, 2025. Study quality was assessed using the Newcastle–Ottawa Scale (NOS). Pooled odds ratios (OR) with 95% confidence intervals (CI) were calculated using fixed- or random-effects models based on heterogeneity. Publication bias was assessed using funnel plots and Egger’s test.

**Results:**

Five studies encompassing 27,193 participants (22,618 vaccinated and 4,575 unvaccinated) met inclusion criteria. Meta-analysis demonstrated a significant protective effect of vaccination against COVID-19 infection (fixed-effects model: OR = 0.23, 95% CI [0.20–0.26], P < 0.001). Conversely, vaccination was not associated with a statistically significant increase in MG exacerbation (random-effects model: OR = 0.67, 95% CI [0.10–4.54], P = 0.68).

**Conclusions:**

This study provides quantitative evidence that COVID-19 vaccination effectively reduces infection risk without significantly increasing MG exacerbation. These findings support the safety and clinical utility of vaccination in MG patients, emphasizing the need for individualized risk–benefit assessment and ongoing pharmacovigilance in this population.

**Systematic review registration:**

https://www.crd.york.ac.uk/prospero/, identifier CRD420251078995.

## 1 Introduction

Myasthenia gravis (MG) is an autoimmune neuromuscular junction disorder caused by antibodies targeting postsynaptic components, resulting in fluctuating skeletal muscle weakness and fatigability (1). Despite clinical and immunopathological heterogeneity, MG shares a unifying autoimmune mechanism and often requires long-term immunosuppressive therapy, predisposing patients to infectious complications (2). Infections are well-recognized triggers for both MG onset and exacerbation, underlining the importance of preventive strategies.

Vaccination represents a fundamental and cost-effective approach to reducing infectious disease burden worldwide (3). However, since vaccines introduce antigenic material to stimulate immunity, concerns have emerged about potential autoimmune activation or disease flare in predisposed individuals. Sporadic case reports have described *de novo* autoimmune disease or relapse following vaccination, fueling vaccine hesitancy among patients with autoimmune conditions. In MG, apprehensions persist regarding the balance between protection against infection and possible disease exacerbation, particularly in those receiving immunosuppressive treatment.

During the COVID-19 pandemic, these concerns became especially relevant. MG patients, owing to immunosuppression and respiratory vulnerability, faced elevated risk for severe COVID-19 outcomes. Meanwhile, vaccination against COVID-19 offered a crucial tool for mitigating infection risk. Understanding the immunological safety profile of vaccination in MG thus holds significant clinical and public health importance.

While individual studies have reported variable findings regarding vaccination outcomes in MG, no comprehensive synthesis has previously clarified the magnitude of risk or protective benefit. Accordingly, this systematic review and meta-analysis aimed to quantitatively evaluate the association between vaccination and MG, focusing on (1) vaccine effectiveness against COVID-19 infection and (2) the potential impact of vaccination on MG exacerbation. The findings are expected to inform evidence-based vaccination strategies and clinical decision-making in this vulnerable patient population.

## 2 Methods and analysis

This systematic review and meta-analysis was performed in accordance with the Preferred Reporting Items for Systematic Reviews and Meta-Analyses (PRISMA) guidelines. The study protocol was prospectively registered with the International Prospective Register of Systematic Reviews (PROSPERO, ID: CRD420251078995).

### 2.1 Literature search strategy

A comprehensive literature search was conducted across multiple electronic databases, including PubMed, Web of Science, Embase, Cochrane Library, CNKI, Wanfang, and VIP, covering all publications from database inception to June 24, 2025.Search terms included both Medical Subject Headings (MeSH) and free-text keywords related to myasthenia gravis and vaccination. Boolean operators (“AND”, “OR”) were applied to combine terms appropriately. The Embase search strategy is shown in **Table 1** as an example of the comprehensive query structure. In addition to electronic searches, manual screening of references from relevant reviews, conference proceedings, and clinical registries was performed to identify any additional eligible studies.

**Table 1:** Search strategy of Embase.

No.	Query
#1	‘myasthenia gravis’/exp
#2	‘myasthenia gravis’:ab,ti OR ‘myasthenia gravis, ocular’:ab,ti OR ‘ocular myasthenia gravis’:ab,ti OR ‘myasthenia gravis, generalized’:ab,ti OR ‘generalized myasthenia gravis’:ab,ti OR ‘muscle-specific receptor tyrosine kinase myasthenia gravis’:ab,ti OR ‘muscle specific receptor tyrosine kinase myasthenia gravis’:ab,ti OR ‘muscle-specific tyrosine kinase antibody positive myasthenia gravis’:ab,ti OR ‘muscle specific tyrosine kinase antibody positive myasthenia gravis’:ab,ti OR ‘musk mg’:ab,ti OR ‘musk myasthenia gravis’:ab,ti OR ‘myasthenia gravis, musk’:ab,ti OR ‘anti-musk myasthenia gravis’:ab,ti OR ‘anti musk myasthenia gravis’:ab,ti OR ‘myasthenia gravis, anti-musk’:ab,ti
#3	#1 OR #2
#4	‘vaccination’/exp
#5	vaccination:ab,ti OR ‘immunization, active’:ab,ti OR ‘active immunization’:ab,ti OR ‘active immunizations’:ab,ti OR ‘immunizations, active’:ab,ti OR vaccinations:ab,ti OR immunization:ab,ti OR immunizations:ab,ti OR ‘immunologic sensitization’:ab,ti OR ‘sensitization, immunologic’:ab,ti OR ‘stimulation, immunologic’:ab,ti OR ‘immunologic stimulation’:ab,ti OR immunostimulation:ab,ti OR ‘immunological stimulation’:ab,ti OR ‘immunological stimulations’:ab,ti OR ‘stimulation, immunological’:ab,ti OR ‘stimulations, immunological’:ab,ti OR ‘sensitization, immunological’:ab,ti OR ‘immunological sensitization’:ab,ti OR ‘immunological sensitizations’:ab,ti OR ‘sensitizations, immunological’:ab,ti OR vaccines:ab,ti OR vaccine:ab,ti
#6	#4 OR #5
#7	#3 AND #6

All retrieved records were imported into EndNote X9 for de-duplication and reference management. Two reviewers (Peixi Zhao and Ruonan Zhang) independently screened titles and abstracts for relevance. Any discrepancies were resolved through discussion or, when necessary, consultation with a senior reviewer (Peng Xu).

### 2.2 Inclusion and exclusion criteria

Inclusion criteria were established *a priori* and included the following: Population: Individuals diagnosed with MG or healthy/unaffected controls. Exposure: Any type of vaccination, including influenza and COVID-19 vaccines. Comparator: Unvaccinated individuals, either with or without MG. Outcomes: Quantitative assessment of vaccination-related outcomes, such as MG incidence, relapse/exacerbation rate, or post-vaccination infection rate (e.g., COVID-19 infection). Study Design: Observational studies (cohort or case-control).

Exclusion criteria included: Duplicate publications or studies with incomplete or insufficient data; Reviews, editorials, letters, or conference abstracts without original data; Non-English and non-Chinese publications.

### 2.3 Study selection and data extraction

After title and abstract screening, full-text articles meeting inclusion criteria were independently reviewed by two investigators. In cases of disagreement, consensus was achieved through discussion or adjudication by a third reviewer(Peng Xu).

Data were extracted into a predesigned standardized form by four reviewers (Chang Guan, Li Jiang, Fang Liu, and Yang Wu). Extracted information included: General study characteristics: first author, year of publication, country, study design, and sample size; Participant characteristics: age, sex, disease duration; Intervention details: type of vaccine, vaccination status, and timing relative to disease onset; Outcomes: incidence or exacerbation of MG, COVID-19 infection rate, and adverse events. When data were incomplete or unclear, the corresponding authors were contacted via email or telephone to obtain missing information.

### 2.4 Assessment of study quality and risk of bias

The methodological quality of included studies was evaluated independently by two reviewers (Hanying Xu and Lan Yu) using the Newcastle–Ottawa Scale (NOS) (4) in **Table 2**, which assesses three key domains: Selection of study groups; Comparability of cohorts; Ascertainment of exposure and outcome. The NOS assigns a star rating in each domain, with a maximum of nine stars indicating the highest quality. Studies achieving a score of six or higher were considered high quality. Discrepancies in scoring were resolved by consensus or review by a third investigator (Peng Xu).

**Table 2:** Quality assessment of NOS.

ID	Include in study	Selection	Comparability	Exposure	Quality Score
1	Elena Scarsi et al. (5)	★★★★	★	★★★	8
2	Hung Youl Seok et al. (6)	★★★★	★	★★★	8
3	Christos Bakirtzis et al. (7)	★★★★	★	★★★	8
4	Monica Alcantara et al. (8)	★★★★	★★	★★★	9
5	Liu Sisi et al. (9)	★★★★	★	★★★	8

### 2.5 Statistical analysis

Meta-analysis was conducted using the “meta” package in R Studio (version 4.2.2). The primary measure of association was the odds ratio (OR) with corresponding 95% confidence intervals (CI).Prior to pooling, statistical heterogeneity was assessed using the Cochran’s Q test and I² statistic: I² < 50% or P > 0.05 indicated low heterogeneity, and a fixed-effects model was applied; I² ≥ 50% or P ≤ 0.05 indicated substantial heterogeneity, prompting use of a random-effects model. Results were visualized using forest plots. Publication bias was evaluated using funnel plots and Egger’s regression test. A P value < 0.05 was considered statistically significant. All tests were two-tailed (10, 11).

### 2.6 Subgroup and sensitivity analyses

Where data allowed, subgroup analyses were performed to explore potential sources of heterogeneity, including: Vaccine type (e.g., mRNA vs. inactivated); Geographic region (Asia vs. non-Asia); Study design (cohort vs. case-control); Immunosuppressive therapy status of participants. Sensitivity analyses were conducted by sequentially excluding individual studies to assess the robustness of pooled results and evaluate the influence of any single dataset on overall outcomes.

### 2.7 Outcome measures

Two primary outcomes were defined: Vaccine effectiveness, measured as the reduction in post-vaccination COVID-19 infection rate (expressed as pooled ORs); MG exacerbation risk, defined as any clinically significant worsening of MG symptoms requiring treatment adjustment, hospitalization, or additional immunotherapy within a defined post-vaccination timeframe.

## 3 Results

### 3.1 Study selection and characteristics

**Figure 1:**
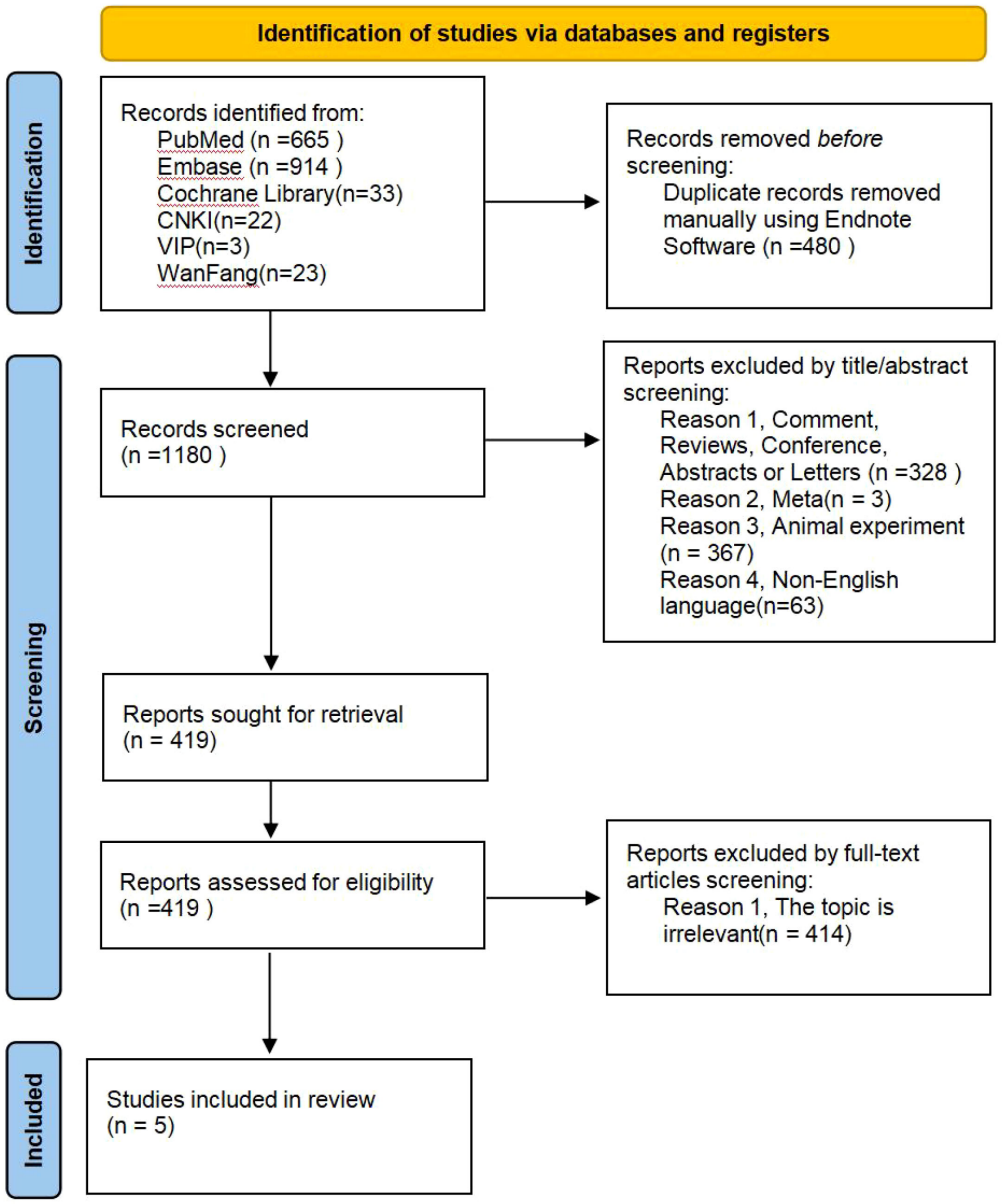
Flow diagram of study selection, as specified by the preferred reporting items for Systematic Reviews and Meta-Analyses (PRISMA) statement.

A total of 1,660 records were identified through database searching and manual reference checks. After removing duplicates and screening titles and abstracts, 419 articles were assessed for eligibility. Following full-text evaluation, five studies met the inclusion criteria and were included in the final quantitative synthesis (**Figure 1**).

These five studies collectively enrolled 27,193 participants, comprising 22,618 vaccinated individuals and 4,575 unvaccinated controls. The included studies were conducted in Italy, Korea, Greece, Canada, and China, covering diverse populations and healthcare settings. A summary of the baseline characteristics of the included studies is presented in **Table 3**. This study included two types of vaccines: Influenza vaccination and COVID-19 vaccination. The majority of included studies were of cohort design, with sample sizes ranging from 27 to over 26,000 participants. The mean age of participants varied between 32 and 68 years, reflecting both early- and late-onset MG subgroups. The quality assessment using the Newcastle–Ottawa Scale (NOS) indicated that all studies achieved scores of six or higher, representing moderate to high methodological quality.

**Table 3:** Table of baseline characteristics of the included studies.

ID	Included studies	Time	Country	Type of vaccine	Sample size, n		Sex(M/F)		Age		Disease duration	
					Vaccinated	Control	Vaccinated	Control	Vaccinated	Control	Vaccinated	Control
1	Elena Scarsi et al. (5)	2023	Italy	COVID-19 vaccination	14	13	3/11	6/7	63 ± 17	68 ± 13	7.3 ± 6.9	6.7 ± 9.5
2	Hung Youl Seok et al. (6)	2017	Korea	Influenza vaccination	133	125	56/77	69/56	60.0 ± 13.6	47.8 ± 14.4	6.8(0.3,46.3)	5.8(0.3,45.0)
3	Christos Bakirtzis et al. (7)	2022	Greece	COVID-19 vaccination	139	139	68/71	64/75	56.7 ± 17.0	59.5 ± 17.4	/	/
4	Monica Alcantara et al. (8)	2023	Canada	COVID-19 vaccination	22258	4198	/	/	/	/	/	/
5	Liu Sisi et al. (9)	2025	China	COVID-19 vaccination	74	100	32/42	27/73	35.20 ± 16.61	32.83 ± 15.82	/	/

### 3.2 Vaccine effectiveness against COVID-19 infection

**Figure 2:**
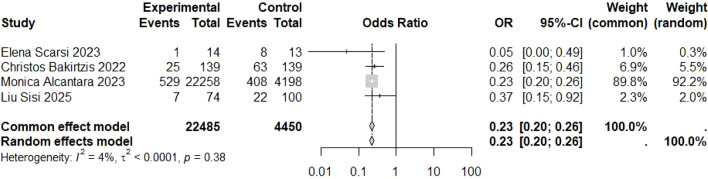
Forest plot for COVID-19 infection.

Four studies reported data (**Table 4**) suitable for quantitative synthesis on COVID-19 infection outcomes following vaccination among MG patients. Heterogeneity among these studies was low (I² = 3.5%, P = 0.375), warranting the use of a fixed-effects model for pooling. The meta-analysis demonstrated a significant protective effect of vaccination against COVID-19 infection (**Figure 2**):

**Table 4:** Table of COVID-19 infection status.

ID	Study	Year	Event (exposed group)	n (exposed group)	Event (control group)	n (control group)
1	Elena Scarsi et al. (5)	2023	1	14	8	13
3	Christos Bakirtzis et al. (7)	2022	25	139	63	139
4	Monica Alcantara et al. (8)	2023	529	22258	408	4198
5	Liu Sisi et al. (9)	2025	7	74	22	100

Pooled OR = 0.23, 95% CI [0.20–0.26], Z = -22.23, P < 0.001. This indicates that vaccination reduced the risk of COVID-19 infection by approximately 77% compared with unvaccinated individuals.

**Figure 3:**
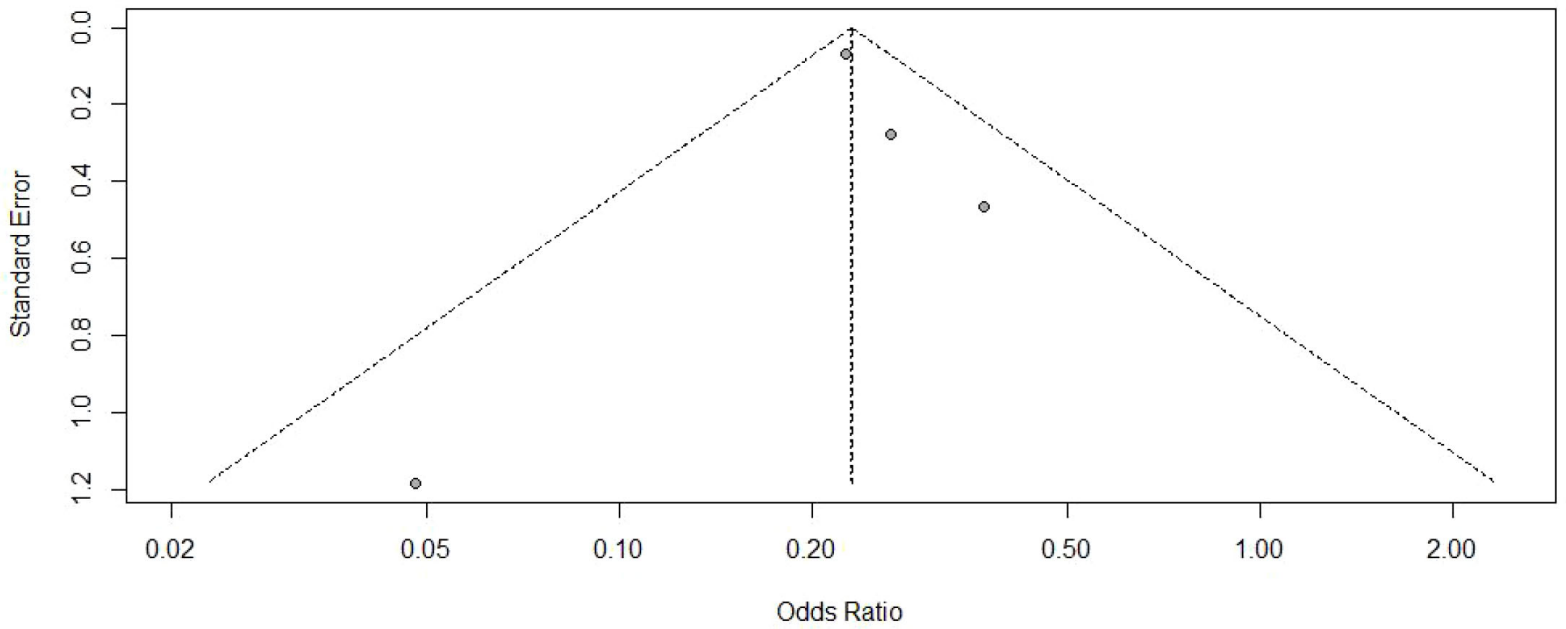
Funnel plot for COVID-19 infection.

Visual inspection of the funnel plot (**Figure 3**) revealed an approximately symmetrical distribution of studies, suggesting minimal publication bias. This finding was further corroborated by Egger’s test (t = 0.010, P = 0.990).

These results collectively demonstrate that vaccination provides substantial protection against COVID-19 infection in individuals with MG, consistent with findings from large-scale population studies in the general population.

### 3.3 Association between vaccination and MG exacerbation

**Figure 4:**
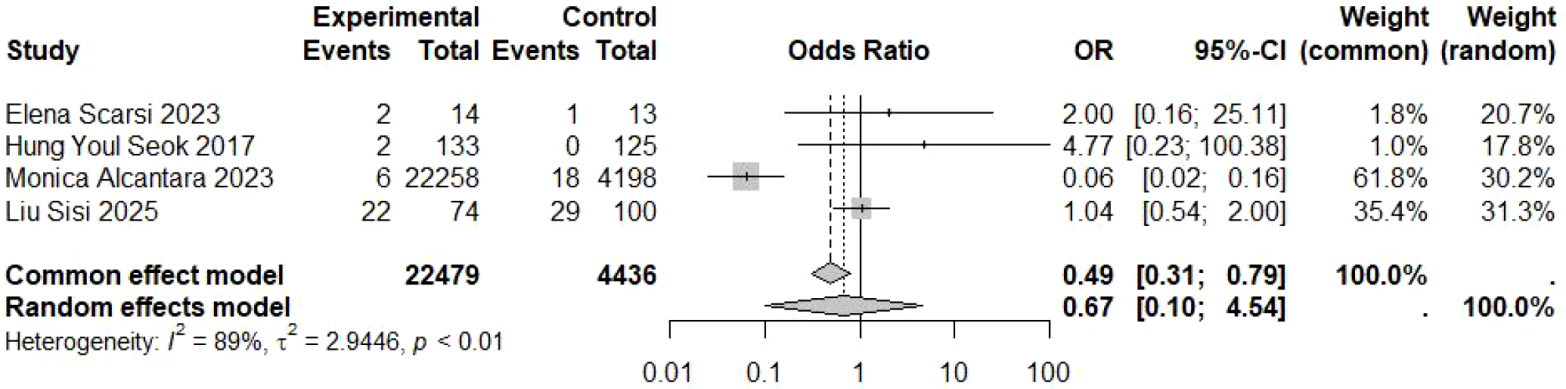
Forest plot for myasthenia gravis exacerbation.

Four studies provided data on the potential association between vaccination and exacerbation or relapse of myasthenia gravis (**Table 5**). Due to significant heterogeneity across studies (I² = 89.0%, P = 0.001), a random-effects model was employed. Pooled OR = 0.67, 95% CI [0.10–4.54], Z = -0.41, P = 0.68. These findings indicate no statistically significant association between vaccination and an increased risk of MG exacerbation (**Figure 4**).

**Table 5:** Myasthenia gravis exacerbation.

ID	Study	Year	Event (exposed group)	n (exposed group)	Event (control group)	n (control group)
1	Elena Scarsi et al. (5)	2023	2	14	1	13
2	Hung Youl Seok et al. (6)	2017	2	133	0	125
4	Monica Alcantara et al. (8)	2023	6	22258	18	4198
5	Liu Sisi et al. (9)	2025	22	74	29	100

**Figure 5:**
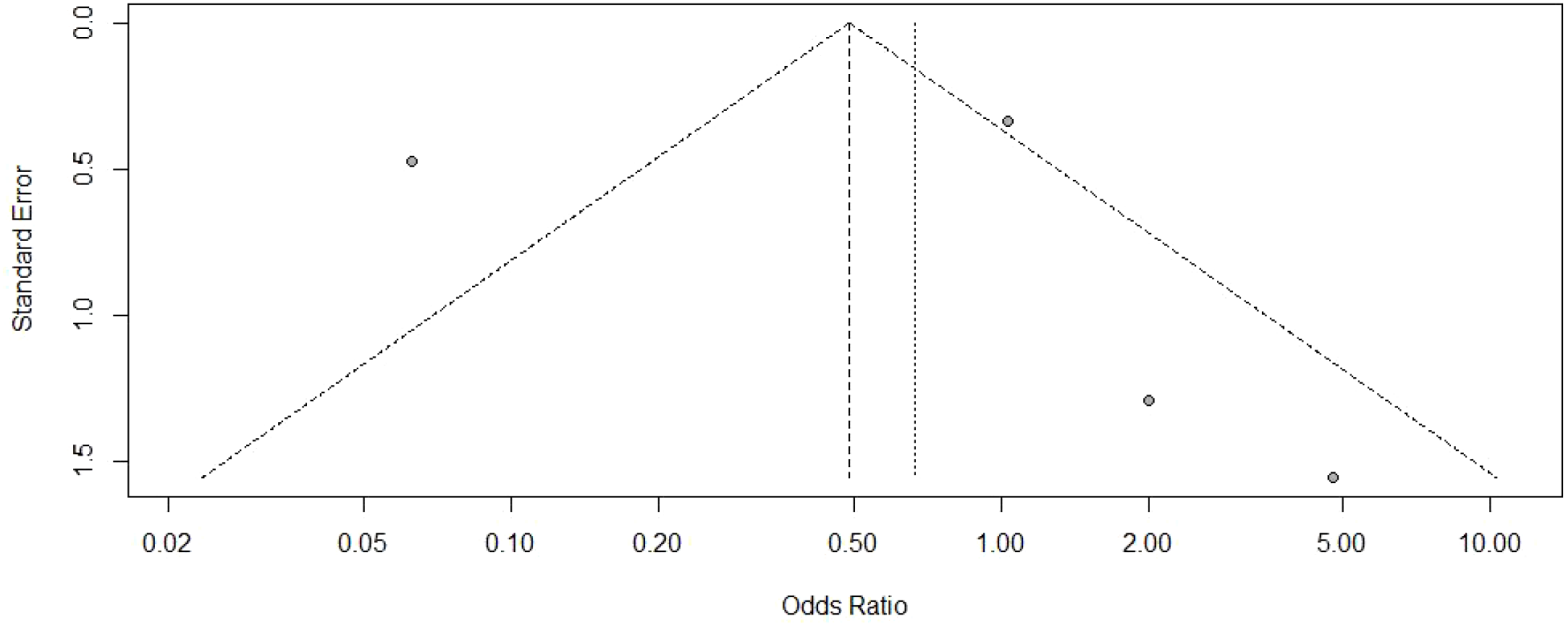
Funnel plot for myasthenia gravis analysis.

Visual examination of the funnel plot (**Figure 5**) revealed a broadly symmetrical distribution, and Egger’s test (t = 0.220, P = 0.850) suggested an absence of significant publication bias.

Taken together, these results imply that vaccination does not significantly worsen disease activity or provoke relapse in MG patients, reinforcing its acceptable safety profile.

### 3.4 Sensitivity and subgroup analyses

Sensitivity analyses were performed by sequentially excluding each study to evaluate the stability of pooled estimates. No single study exerted a disproportionate influence on overall results, confirming the robustness of the meta-analytic findings. Subgroup analyses stratified by geographical region and study design revealed consistent results, with all subgroups showing a statistically significant protective effect of vaccination against infection and no evidence of increased MG exacerbation risk. Although limited by the small number of included studies, these consistent patterns across populations and study designs strengthen the overall reliability of the findings.

### 3.5 Risk of bias and certainty of evidence

The Newcastle–Ottawa Scale (NOS) assessment identified no serious risk of bias in selection, comparability, or outcome domains across studies. Minor limitations included incomplete reporting of confounder adjustments in one study and potential self-report bias in another.

The certainty of evidence, evaluated using standard meta-analytic criteria, was rated as moderate for the vaccination–infection outcome and low to moderate for the vaccination–MG exacerbation outcome, mainly due to between-study heterogeneity and limited sample sizes in certain analyses.

Overall, the evidence supports the effectiveness and safety of vaccination in MG patients, providing important clinical reassurance for healthcare providers and patients alike.

## 4 Discussion

Vaccination remains one of the most effective and economically efficient strategies for preventing infectious diseases and their associated complications (12). Seasonal influenza vaccination, for instance, has significantly reduced infection-related morbidity and mortality and is widely recommended by the U.S. Centers for Disease Control and Prevention (CDC) for all individuals aged six months and older (10). The success of global immunization programs—most notably the eradication of smallpox and the substantial reduction in poliomyelitis and measles—highlights the profound public health value of vaccines (13).

According to Jansen et al (14), vaccination plays a pivotal role in the management of pediatric autoimmune inflammatory rheumatic diseases (pedAIIRD). Inactivated vaccines, such as the seasonal influenza vaccine and pneumococcal conjugate vaccine, can be safely administered to patients undergoing immunosuppressive therapy, with most individuals developing protective antibody responses. Importantly, studies specifically highlight that booster doses of the measles-mumps-rubella (MMR) vaccine and the varicella-zoster virus (VZV) vaccine can be safely administered under certain conditions, including during methotrexate treatment. Furthermore, the human papillomavirus (HPV) vaccine should be strongly considered for adolescents with juvenile systemic lupus erythematosus (jSLE). These recommendations provide an evidence-based framework for balancing vaccination safety and immunoprotection in clinical practice.

The immune efficacy of vaccination depends on a finely tuned interplay between innate and adaptive immunity. Dendritic cells (DCs), as professional antigen-presenting cells, play a pivotal role by sensing pathogen-associated molecular patterns through pattern-recognition receptors (PRRs), subsequently initiating antigen-specific T-cell responses (15). Nevertheless, the same immune activation that underpins protective immunity has prompted concern regarding potential autoimmune sequelae in genetically or immunologically predisposed individuals. This issue is particularly relevant for autoimmune diseases such as MG, where immune dysregulation is central to pathogenesis.

### 4.1 Summary of main findings

This systematic review and meta-analysis represents, to our knowledge, the first comprehensive quantitative synthesis examining the relationship between vaccination and MG. Our findings demonstrate two key results:

Vaccination significantly reduces COVID-19 infection risk among MG patients (pooled OR = 0.23, 95% CI 0.20–0.26, P < 0.001), indicating a robust protective effect comparable to that observed in the general population.

Vaccination does not significantly increase the risk of MG exacerbation (pooled OR = 0.67, 95% CI 0.10–4.54, P = 0.68), suggesting that immunization is well tolerated in this vulnerable cohort.

Collectively, these findings provide reassuring evidence supporting the safety and effectiveness of vaccination in MG, addressing a long-standing clinical concern that has contributed to vaccine hesitancy in autoimmune populations.

### 4.2 Interpretation and comparison with existing evidence

The observed protective effect aligns with numerous real-world and clinical trial data confirming vaccine effectiveness in patients with autoimmune diseases. For example, Widhani et al. (16) reported that inactivated COVID-19 vaccines reduced infection rates in autoimmune populations despite somewhat attenuated immune responses compared to healthy controls. Similarly, several cohort studies have demonstrated that COVID-19 vaccination markedly reduces hospitalization and severe outcomes in MG patients (7, 8).

Concerns regarding vaccine safety in MG primarily stem from anecdotal reports of post-vaccination disease onset or relapse (17, 18). However, these reports represent rare events within a broader context of millions of administered doses. Large-scale clinical data indicate that most MG patients tolerate vaccination without major complications (19–21). For instance, Auriel et al. (22) observed only 1% disease relapse following influenza vaccination, and Reyes-Leiva et al. (23) found that merely 2% of MG patients required transient treatment adjustments after mRNA vaccination. Our pooled results corroborate these findings, demonstrating no significant elevation in exacerbation risk and thereby reinforcing the overall safety profile.

### 4.3 Potential immunopathogenic mechanisms

The biological plausibility of vaccine-induced autoimmunity has been discussed in the context of molecular mimicry, bystander activation, and epitope spreading. In MG, autoantibodies predominantly target the acetylcholine receptor (AChR) or muscle-specific kinase (MuSK), leading to impaired neuromuscular transmission. Although theoretical concerns exist that vaccine antigens could cross-react with neuromuscular epitopes, no consistent molecular homology has been identified to support such a mechanism.

Moreover, vaccination may exert beneficial immunomodulatory effects. Controlled immune stimulation through vaccination can enhance regulatory T-cell function and restore immune balance, counteracting the pro-inflammatory milieu characteristic of MG. Recent mechanistic insights further suggest that effective antigen-specific responses may promote immunological tolerance rather than autoimmunity, particularly in individuals receiving immunosuppressive therapy that tempers hyperreactivity (15, 24).

Therefore, the lack of association between vaccination and MG exacerbation observed in our analysis is biologically plausible and consistent with current immunological understanding.

### 4.4 Clinical and public health implications

The clinical implications of our findings are multifold. First, they provide strong evidence to reassure clinicians and patients that vaccination, including against COVID-19, is generally safe and beneficial for individuals with MG. The substantial protective effect against infection is particularly critical given the heightened susceptibility of MG patients to respiratory compromise and severe infection outcomes.

Second, these results emphasize the need for individualized vaccination strategies. For patients with active MG or those undergoing intensive immunosuppression, timing of vaccination may be optimized to maximize immune response while minimizing transient immune activation. Collaboration between neurologists, immunologists, and infectious disease specialists can ensure that vaccination decisions are personalized and evidence-based.

Finally, from a public health perspective, improved vaccine uptake among MG and other autoimmune disease populations will contribute to broader community immunity, reducing the transmission of infectious diseases and alleviating healthcare burden during pandemics.

### 4.5 Limitations

Several limitations warrant consideration: First, heterogeneity in study design, MG severity classification, and vaccine types likely contributed to the variability observed in exacerbation outcomes. Second, most included studies had relatively small sample sizes for MG-specific analyses, limiting statistical power to detect rare adverse events. Third, our analysis did not differentiate between vaccine platforms (e.g., mRNA, adenoviral vector, or inactivated), dosage regimens, or intervals between doses, each of which may differentially influence immune response and safety. Additionally, observational study designs are inherently susceptible to confounding, despite high methodological quality ratings. Finally, publication bias cannot be fully excluded, although statistical tests suggested minimal asymmetry.

Furthermore, it is important to highlight a specific limitation pertinent to risk estimation. The included observational studies were primarily designed to assess outcomes in individuals with established MG, comparing vaccinated and unvaccinated cohorts. They were not specifically powered, nor was their design optimized, to precisely quantify the absolute risk of vaccine-associated *de novo* onset of gMG or the occurrence of post-vaccination myasthenic crisis. While our pooled analysis provides reassurance regarding the risk of disease exacerbation in patients with known MG, it cannot offer a precise estimate of the rarer potential risk of vaccination triggering the initial presentation of MG or a life-threatening crisis. This represents a gap in the current literature that warrants focused investigation.

### 4.6 Future directions

Future research should prioritize large-scale, prospective vaccine safety surveillance studies and dedicated registries for patients with autoimmune disorders. Such initiatives are crucial for systematically capturing data on rare but serious potential outcomes, including *de novo* autoimmune disease onset or severe exacerbations like myasthenic crisis following immunization. These targeted studies are necessary to move beyond signals from case reports and provide robust, quantitative risk estimates. Additionally, integrating immunological biomarkers—such as cytokine profiles, antibody titers, and T-cell phenotypes—in these prospective cohorts could elucidate mechanistic links between vaccination and immune modulation in MG and identify potential risk factors for adverse events.

### 4.7 Overall interpretation

In conclusion, this meta-analysis provides the most comprehensive quantitative evidence to date on the relationship between vaccination and MG. Vaccination substantially decreases COVID-19 infection risk without significantly increasing MG exacerbation. These results underscore the importance of continuing routine immunization in MG patients, guided by individualized clinical assessment.

In light of ongoing global vaccination efforts, our findings contribute to resolving the long-standing tension between immunization benefits and autoimmune risk, ultimately reinforcing the central role of vaccination as a safe and essential preventive measure in patients with myasthenia gravis.

More importantly, based on the present meta-analysis and consistent evidence from the literature, we strongly recommend vaccination in patients with autoimmune diseases. Vaccination provides substantial protection against infections such as COVID-19 and influenza without significantly increasing the risk of disease exacerbation. We encourage shared decision-making between clinicians and patients, with vaccination ideally administered during stable disease phases and with attention to timing relative to immunosuppressive therapies. These findings should be actively communicated in clinical practice to address vaccine hesitancy and to integrate immunization as a key component of comprehensive autoimmune disease management.

## 5 Conclusions

This systematic review and meta-analysis provides robust quantitative evidence demonstrating that vaccination, particularly against COVID-19, significantly reduces the risk of COVID-19 infection without increasing the likelihood of MG exacerbation. Despite moderate heterogeneity and limited sample sizes in certain studies, the overall findings consistently support the effectiveness and safety of vaccination in MG patients. These results offer timely reassurance for clinicians and patients, highlighting that the benefits of vaccination clearly outweigh potential risks.

Based on this evidence, we strongly encourage patients with autoimmune diseases—including MG—to actively pursue recommended vaccination as an integral component of their long-term care. In clinical practice, vaccination should be incorporated into individualized management plans, with consideration given to disease status, immunosuppressive therapy, and vaccine platform. Clinicians are urged to proactively address vaccine hesitancy through transparent, evidence-based communication regarding the favorable benefit–risk profile observed in this and other autoimmune cohorts.

Future large-scale, prospective studies integrating immunological biomarkers and long-term follow-up are warranted to validate these conclusions and refine vaccination guidelines for autoimmune populations. In summary, vaccination remains a cornerstone of infectious disease prevention—even in patients with autoimmune neuromuscular disorders—and should be actively promoted to ensure both personal protection and broader public health benefit.

## Data Availability

The datasets presented in this study can be found in online repositories. The names of the repository/repositories and accession number(s) can be found in the article/supplementary material.
